# RELATIONSHIP BETWEEN ALEXITHYMIA AND PARKINSON’S DISEASE IN A TUNISIAN SAMPLE

**DOI:** 10.1192/j.eurpsy.2023.2034

**Published:** 2023-07-19

**Authors:** N. Messedi, E. Fakhfakh, S. Kolsi, I. Chaari, F. Charfeddine, L. Aribi, N. Farhat, C. Mhiri, J. Aloulou

**Affiliations:** 1 Psychiatry Department B, Hedi Chaker Hospital Sfax, Tunisia; 2 neurology department, Hbib Bourguiba Hospital Sfax, Tunisia, Sfax, Tunisia

## Abstract

**Introduction:**

Several psychiatric signs are part of non-motor signs of parkinson’s disease (PD), including alexithymia.

**Objectives:**

The objective of this study is to determine the frequency of alexithymia in patients with PD and to study factors associated with it.

**Methods:**

Descriptive and analytical cross-sectional study collected from patients followed at the neurology consultation of Habib Bourguiba’s University Hospital in Sfax, Tunisia. We used:

- A sociodemographic, clinicaland therapeutic datasheetincludingthe Hoehn and Yahr motor scalefor the staging of the functional disability associated with PD
- The Toronto Alexithymia Scale (TAS-20) with a cutoff score = 61

**Results:**

We recruited 47 patients. The average age was 61.47 years with a sex ratio (M/W) = 1.47. The average age of onset of the disease was 51.97 years. Sleep disorders were present in 51.1% of cases.41 patients (87.23%) were treated with dopa therapy. An Hoehn and Yahr stage ≥ 3 was found in 25.5% of patients.

TAS: The mean score was 47.38 and alexithymia frequency was 19.1%.

Alexithymia was statistically correlated with the presence of sleep disorders (P=0.023) and with an Hoehn and Yahr stage ≥ 3 (p=0.039).The occurrence of alexithymia was not significantly associated with taking dopatherapy (P= 0.31).

**Conclusions:**

Alexithymia has been quite frequent in patients with PD and associated with motor gravityand sleep disorders. It is considered as a non-motor symptom of the disease that needs to be treated promptly.

**Disclosure of Interest:**

None Declared

